# Chronic obstructive pulmonary disease with short-acting inhaled pharmacotherapy increases the risk of prostate cancer: A two-stage database approach

**DOI:** 10.1371/journal.pone.0203377

**Published:** 2018-09-06

**Authors:** Hui-Wen Lin, Li-Fong Lin, Hung-Chou Chen, Tsan-Hon Liou, Shih-Wei Huang

**Affiliations:** 1 Department of Mathematics, Soochow University, Taipei, Taiwan; 2 Evidence-Based Medicine Center, Wan Fang Hospital, Taipei Medical University, Taipei, Taiwan; 3 Department of Physical Medicine and Rehabilitation, Shuang Ho Hospital, Taipei Medical University, Taipei, Taiwan; 4 Institute of Gerontology and Health Management, Taipei Medical University, Taipei, Taiwan; 5 Department of Physical Medicine and Rehabilitation, School of Medicine, College of Medicine, Taipei Medical University, Taipei, Taiwan; 6 Graduate Institute of Sports Science, National Taiwan Sport University, Taoyuan, Taiwan; University of Bern, SWITZERLAND

## Abstract

**Background:**

Patients with chronic obstructive pulmonary disease (COPD) are at a higher risk of many types of cancer. However, specific investigation of the risk of prostate cancer and the influence of COPD pharmacotherapy in patients with COPD is lacking. This study investigated the risk and influence of COPD pharmacotherapy on risk of prostate cancer in patients with COPD.

**Methods:**

This retrospective cohort study used data from Taiwan’s Longitudinal Health Insurance Database 2005 (LHID2005). The study cohort comprised COPD patients who received treatment between 2004 and 2008, and who were identified from the LHID2005. The control cohort comprised patients without COPD and was matched to the study cohort by age and sex. Two-stage propensity score calibration with the National Health Interview Survey 2005 was performed to obtain the missing confounders of smoking, alcohol drinking, and body mass index in the LHID. The hazard ratio (HR) and adjusted HR were estimated. Moreover, the influence of inhaled medications and other related medication on the risk of prostate cancer was analyzed by Cox proportional hazard regression.

**Results:**

The COPD cohort comprised 12,774 patients, and the control cohort comprised 38,322 patients (1:3). The incidence of prostate cancer was 633 per 100,000 person-years in the COPD cohort and 361 per 100,000 person-years in the control cohort. The propensity score calibration-adjusted HR was 1.62 (95% CI, 1.40–1.87, p < 0.001) in the COPD cohort. Further analysis revealed that the adjusted HR for the risk of prostate cancer was 1.61 (95% CI, 1.19–2.16, p = 0.002) in patients with COPD who used short-acting muscarinic antagonists (SAMAs) and 1.89 (95% CI, 1.40–2.54, p < 0.001) in patients with COPD who used short-acting beta-agonists (SABAs). COPD patients had lower risk of prostate cancer when using statin (HR = 0.63, 95% CI, 0.45–0.89, p = 0.010) or aspirin (HR = 0.55, 95% CI, 0.35–0.85, p = 0.008).

**Conclusion:**

Patients with COPD are at a higher risk of prostate cancer, particularly those using SAMAs or SABAs. This finding suggests that inflammation control may be an effective strategy for decreasing the risk of prostate cancer.

## Introduction

Chronic obstructive pulmonary disease (COPD) is a chronic progressive disease characterized by pulmonary function decline and persistent airway inflammation; its global prevalence is 5%–13%.[1, 2] It is also one of the most prevalent causes of mortality and is expected to have the third highest disease burden globally by 2020.[3, 4] Episodes of acute exacerbation are common among patients with COPD, and lead to pulmonary function decline, increased mortality and morbidity, and disability; they also increase the economic burden of health care expenditures faced by society.[5–8] In addition to the pulmonary system, COPD can affect nonrespiratory systems.[9, 10] For example, some previous studies have demonstrated that COPD patients are at a risk of lung cancer.[11–13] Another recent study reported that COPD can increase the risk of several extrapulmonary cancers.[14] These results suggest that COPD is associated with cancer; furthermore, patients with COPD are hypoxemic and exhibit systemic inflammation, which provide an advantageous environment for cancer development.[11, 15]

Prostate cancer is a slow and progressive disease, and is one of the most common cancers among men in Western countries.[16] Previous research has described many etiologies for prostate cancer, such as aging, family history, race, androgen, obesity, and chronic prostate inflammation.[17–19] In addition to local inflammation of the prostate, some recent studies have reported that systemic inflammation can induce the carcinogenesis of prostate cancer.[20, 21] Toriola et al. conducted a prospective cohort study with an average follow-up period of 24 years in Finland; they investigated the association of three common inflammatory markers (C-reactive protein, fibrinogen, and leukocyte count) with the risk of prostate cancer, and found that men with elevated leukocyte counts had a 2.57-fold increased risk of prostate cancer mortality.[20] Another previous study compared a single-nucleotide polymorphism between normal individuals and patients with prostate cancer, and determined that TLR1, TLR6, OAS1, and OAS2 genes, which are associated with innate inflammation pathways, were nominally associated with the risk of advanced prostate cancer.[21]

Both COPD and prostate cancer are chronic progressive diseases and critical public health challenges. As described earlier, inflammation is one of the factors contributing to the carcinogenesis of prostate cancer, and COPD is characterized by inflammation. A recent population-based study investigated a wide spectrum of cancer risk among patients with COPD, and revealed that these patients exhibited a 1.20-fold higher risk of prostate cancer than controls; however, risk factors such as smoking and obesity were not controlled for in that study.[22] A recent systemic review and meta-analysis indicated glutathione S-transferase T 1 (GSTT1) and glutathione S-transferase M 1 (GSTM1) polymorphisms are associated with increased risk of prostate cancer in Asians.[23] Thakur et al investigated the role of GSTM1 and GSTT1 null genotypes as risk factors for COPD and prostate cancer and found null genotype of GSTT1 2 fold significantly increased risk for COPD.[24] Moreover, the influence of inhaled pharmacotherapy used by patients with COPD on the risk of prostate cancer has not been analyzed. Therefore, we conducted a retrospective cohort study to investigate whether COPD is a risk factor for prostate cancer, and analyzed the effect of inhaled medication on the risk of prostate cancer in such patients.

## Methods

### Study data

Data for this study was collected from Taiwan’s Longitudinal Health Insurance Database 2005 (LHID2005), which contains the claims data of 1 million residents of Taiwan; these residents account for approximately 5% of the total number of beneficiaries and were randomly selected from the insurants of the National Health Insurance (NHI) program of Taiwan. The NHI program, established on March 1, 1995, provides comprehensive medical care service coverage for more than 99% of residents of Taiwan. All NHI claims data is included in the National Health Insurance Research Database (NHIRD), which is maintained by the National Health Research Institutes and contains registration files and claims data for reimbursement. For research purposes and improving the quality of care in Taiwan, the LHID2005 was derived from the NHIRD. The LHID2005 contains information on age, sex, diagnoses made according to the International Classification of Diseases, Ninth Revision, Clinical Modification (ICD-9-CM) codes, medication prescriptions, dosages, duration of medication usage, medical interventions (e.g., surgery), and medical expenditures.

### Validation of the study data

Because the LHID2005 contains no information on body mass index (BMI), smoking, or alcohol drinking, which are important variables that affect patients with COPD and prostate cancer, we used the National Health Interview Survey (NHIS) database to acquire these variables as an external validation study. The NHIS is a nationwide cross-sectional health survey, and the NHIS2005 was conducted from April to August 2005 on residents of Taiwan by using a stratified sampling scheme. Detailed information on the NHIS is available in a previous study by Guo et al.[22]

### Patients and study design

From January 1, 2004, to December 31, 2008, we conducted a longitudinal, retrospective, case–control cohort study to investigate whether COPD is a risk factor for prostate cancer. Both the study and control cohorts were identified from the LHID2005. The study cohort (COPD cohort) comprised patients with COPD who had records of ambulatory care visits for COPD treatment from January 1, 2004 to December 31, 2004. The COPD cohort comprised male patients who were older than 50 years, had had at least two consecutive diagnoses of COPD (ICD-9-CM codes 491, 492, and 496) before receiving COPD treatment in 2004, had at least three records of COPD pharmacotherapy in 2004, received stationary medication (no medication change) for COPD within the year of treatment initiation, and had no record of COPD medication 1 year before initiating treatment. The exclusion criteria of the study cohort were records of diagnostic codes for prostate cancer before 2004 and missing data in the LHID. The control cohort consisted of the remaining patients from the LHID2005, and was matched to the study cohort by age and sex in a 3:1 ratio. All patients enrolled in this study were followed up until the occurrence of prostate cancer, death, withdrawal from the NHI program (migration to another country or refusal to pay the fee of insurance), or December 31, 2008. The outcome of this study was prostate cancer development (ICD-9-CM code 185.XX) during the 4-year longitudinal follow-up period. Because the NHI program defines prostate cancer as a catastrophic illness, cancer-associated medical expenditures were waived. For catastrophic illness certification, in addition to clinical presentation and image evaluation, the diagnosis of prostate cancer depends on pathological proof. Thus, the diagnostic accuracy of prostate cancer is reliable in this study. The NHIRD deidentifies and encrypts data, thus ensuring patient privacy and that researchers cannot trace individual patients or health service providers by using the data. Therefore, this study was exempted from a complete review by any institutional review board.

### Covariates of both cohorts

To determine the possible confounders related to the risk of prostate cancer, we calculated the Charlson Comorbidity Index (CCI) by following the comorbidities of both cohorts in this study: myocardial infarction (ICD-9-CM codes 410 and 412), congestive heart failure, peripheral vascular disease, cerebral vascular disease, dementia, rheumatic diseases (rheumatoid arthritis, ICD-9-CM code 714.0; systemic lupus erythematosus, ICD-9-CM code 710.0), peptic ulcer, liver diseases, renal disease, diabetes, metastatic carcinoma, and acquired immune deficiency syndrome.

### Medication and inhaled pharmacotherapy for COPD

Using the LHID2005, we analyzed the use of the following medications in COPD patients: aspirin, statin, oral steroid, nonsteroidal anti-inflammatory drug (NSAID), and inhalation medicine. The prescribed inhalation medication was classified as follows: (1) short-acting muscarinic antagonists (SAMAs): ipratropium bromide metered-dose inhaler (MDI) (Atrovent, Boehringer Ingelheim); (2) short-acting β-agonists (SABAs): fenoterol hydrobromide MDI (Berotec, Boehringer Ingelheim), terbutaline sulfate Turbuhaler (Bricanyl, GlaxoSmithKline), or salbutamol sulfate MDI (Ventolin, GlaxoSmithKline); (3) long-acting muscarinic antagonists (LAMAs): tiotropium HandiHaler (Spiriva, Boehringer Ingelheim); (4) long-acting β-agonists (LABAs): formoterol Turbuhaler (Oxis, AstraZeneca) or salmeterol MDI (Serevent, GlaxoSmithKline); and (5) LABAs plus inhaled corticosteroids (ICSs): formoterol plus budesonide Turbuhaler (Symbicort, AstraZeneca) or salmeterol plus fluticasone MDI (Seretide, GlaxoSmithKline). The accumulated prescription duration for each claim of these inhaled pharmacotherapies must be longer than 14 days. For patients treated with regular long-acting inhalation medication (LAMAs, LABAs, or LABAs plus ICSs), the short-term use of SABAs or SAMAs as an on-demand rescue for acute exacerbation was allowed. Notably, patients with COPD using a combination of inhalation medication were excluded from this analysis. In addition to inhaled medicine, other medication such as aspirin, oral form of steroid, nonsteroidal anti-inflammatory drugs (NSAIDs), and the statins (included simvastatin (ATC code C10AA01), lovastatin (ATC code C10AA02), pravastatin (ATC code C10AA03), fluvastatin (ATC code C10AA04), and atorvastatin (ATC code C10AA05)) were analyzed in this study.

### Statistical analyses

**Fig 1:**
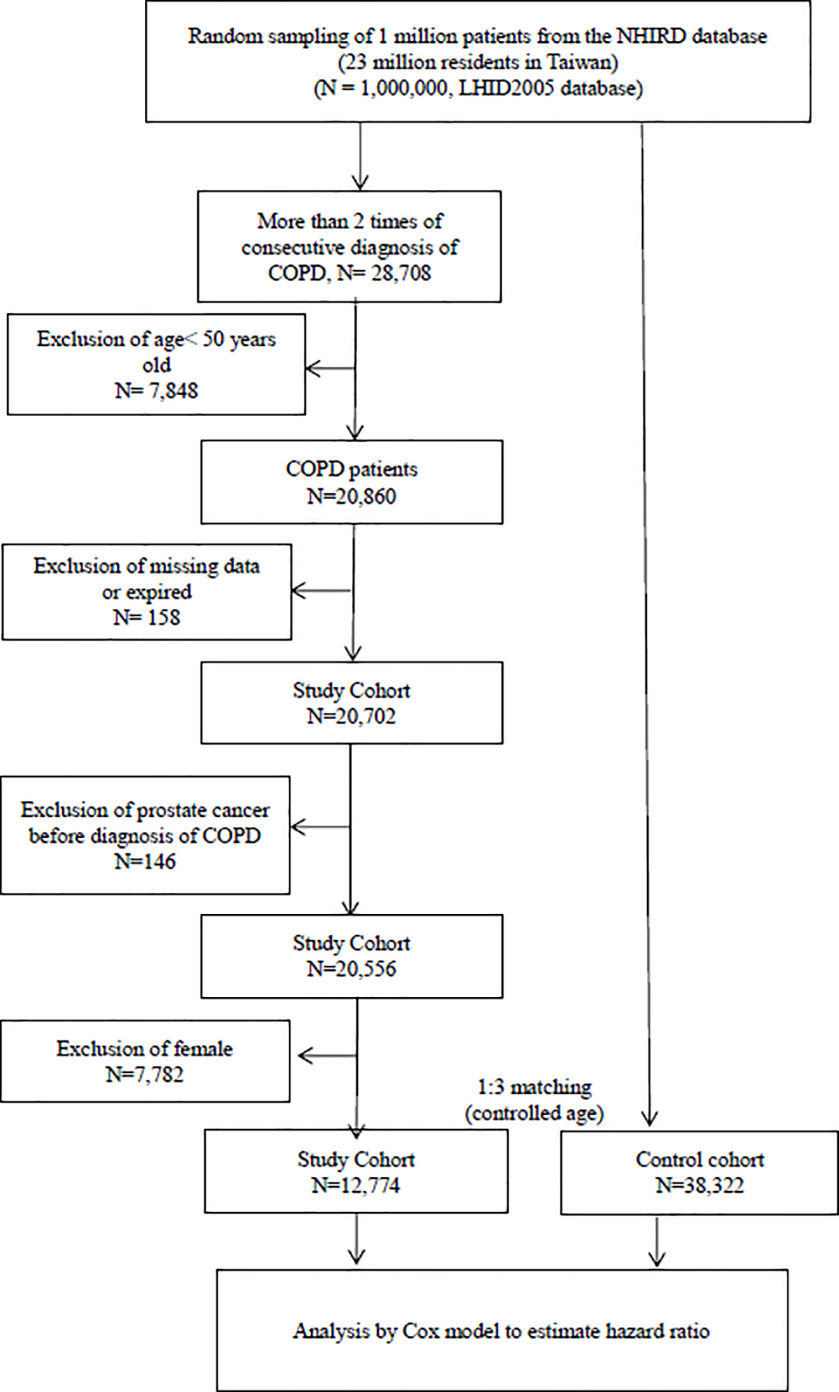
Study flowchart.

All statistical analyses were performed using the SAS statistical package (version 9.1.3; SAS Institute, Cary, NC, USA). A p value of <0.05 was considered statistically significant. The demographic data and comorbidities were analyzed using Pearson’s chi-square test. As noted earlier, essential demographic data for prostate cancer, such as BMI, smoking, and alcohol drinking, could not be acquired from the main database (LHID2005). Based on the two-stage design, we combined patients from the main and validating databases to adjust for these missing confounders.[25] From the LHID2005, 12,774 patients with COPD were included in the study cohort, and 38,322 patients without COPD were included in the control cohort. (Fig 1) Another 312 patients with COPD and 804 patients without COPD were identified from the NHIS for the validation cohort. The incidence and hazard ratio (HR) were analyzed using a stratified Cox model to determine the risk of prostate cancer in the COPD cohort, in comparison with that in the control cohort. All demographic data, comorbidity, and COPD medication variables were analyzed in this model to calculate the adjusted HR for subsequent prostate cancer development. We also compared the demographic data and comorbidities of patients with or without prostate cancer in the COPD cohort by using Pearson’s chi-square test. Finally, we used multivariable Cox proportional hazard regression to analyze the effect of COPD medication on the risk of prostate cancer in patients with COPD.

### Two-stage propensity score calibration approach

To determine the influence of the missing confounders of BMI, smoking, and alcohol drinking in the LHID2005, we adopted a two-stage approach that has been widely utilized to integrate different databases.[25, 26] Therefore, in this two-stage approach, which was described by Stürmer et al.,[26] the propensity score was used to adjust for the missing confounders acquired from the NHIS2005. The detailed statistic method procedures as follows: suppose that D denotes a COPD indicator variable. A value of 1 is selected for patients with COPD and 0 for patients without COPD. Let E be a matrix of observed confounders, namely age, sex, and Charlson Comorbidity Index (CCI). Let M denote an indicator for the missing confounders, smoking, alcohol consumption, and BMI.

1. Let the probability PS = Pr (D = 1|E) be a propensity score in the main study.
2. The propensity score PS_c_ = Pr(D = 1|*E*) is defined as the error-prone variable, and PS_*m*_ = Pr(*D* = 1|*E*, *M*) is defined as the gold standard in the validation study.
3. prostate cancer association in a Cox proportional hazard model is defined as H(t|D,PS) = H_0_(*t*) exp{*βD* + *β*_*c*_*PS*}.
4. The measurement error in the model is given by E(PS_*m*_|*D*, PS_*c*_) = *γ*_0_ + *γD* + *γ*_*c*_PS_*c*_, The estimation of the parameter (*γ*_0_,*γ*_*c*_,*γ*) can be obtained by the least squares method of the regression equation.
5. Both LHID and NHIS datasets from this study were sampled from the same population, the regression calibration after adjustment for E and M confounding factor estimator for the effect of E is given by $\hat{\beta^{*}}=\hat{\beta }-\Delta \hat{\gamma }$, where $\Delta =\frac{\hat{\beta_{c}}}{\hat{\gamma_{c}}}$. The parameter estimator ($\hat{\beta },\hat{\beta_{c}}$) can be obtained by step 3 Cox regression, and ($\hat{\gamma },\hat{\gamma_{c}}$) can be obtained by step 4 regression model.

The term $\hat{\beta^{*}}$ is estimated because of failure to adjust for missing confounders.

### Bootstrap method for sensitively analysis

We use bootstrap procedures for sensitively analysis, the bootstrap method simply resample from the empirical distribution. We present a formal algorithm. Let B denote the number of bootstrap replications. $\hat{\beta }$ be a estimator of β.

1. Set B = 1000, j = 1.
2. j ≤ B, then do 2 – 5.
3. Let S* be the realization of a random sample drawn from study samples with replacement.
4. Let $\hat{\beta }*=\hat{\beta }(S^{*})$ be a estimator from Cox model with sample S*.
5. Let j = j+1
6. Let ${\hat{\beta }}_{\left(1\right)}^{*}\leq {\hat{\beta }}_{\left(2\right)}^{*}\leq \ldots \leq {\hat{\beta }}_{\left(B\right)}^{*}$ denote the ordered of $\hat{\beta }*$. The percentile bootstrap 95% confidence interval for β is (${\hat{\beta }}_{\left(2.5\%*B\right)}^{*},{\hat{\beta }}_{\left(97.5\%*B\right)}^{*}$).

**Fig 2:**
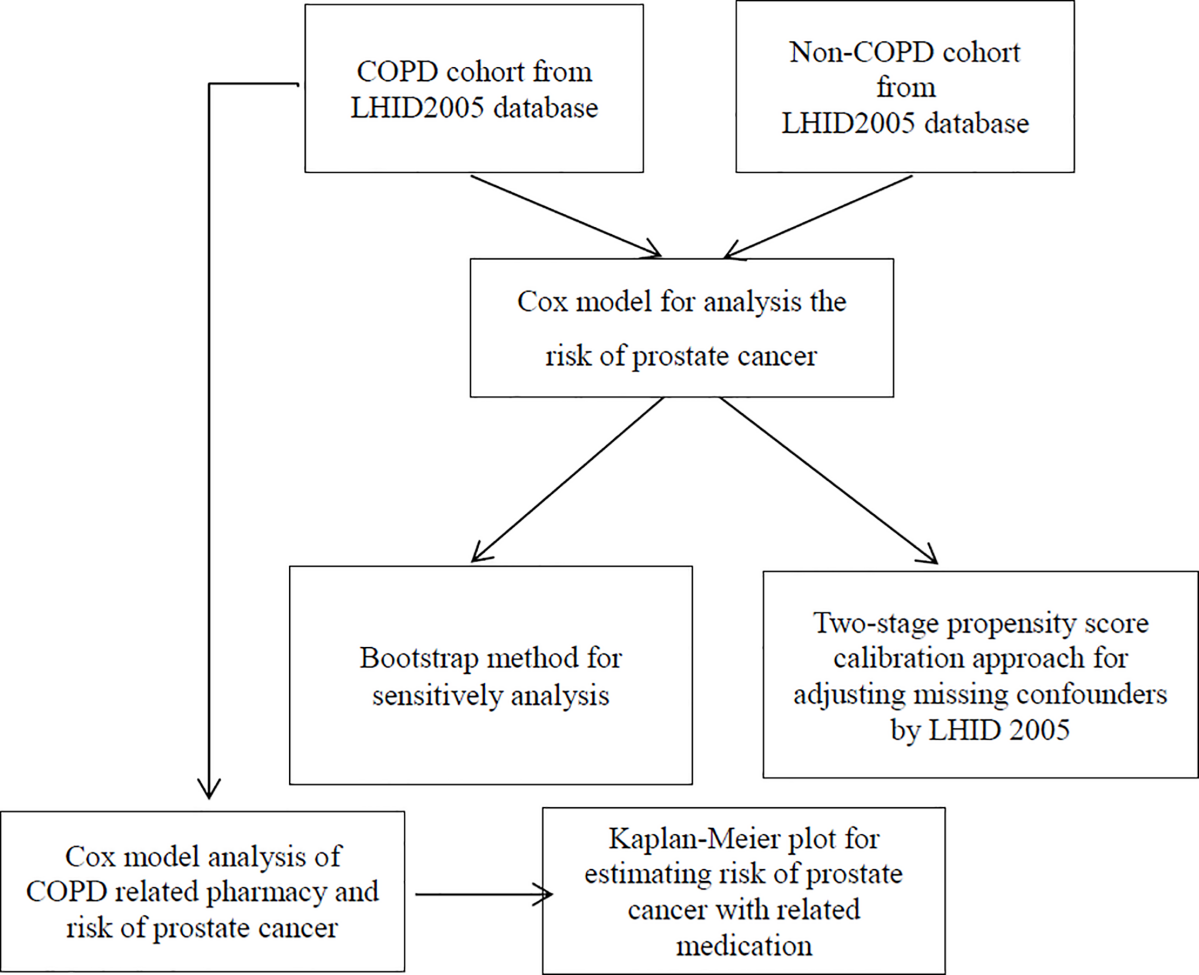
Data analysis framework.

The framework of data analysis is presented as Fig 2.

## Results

**Fig 3:**
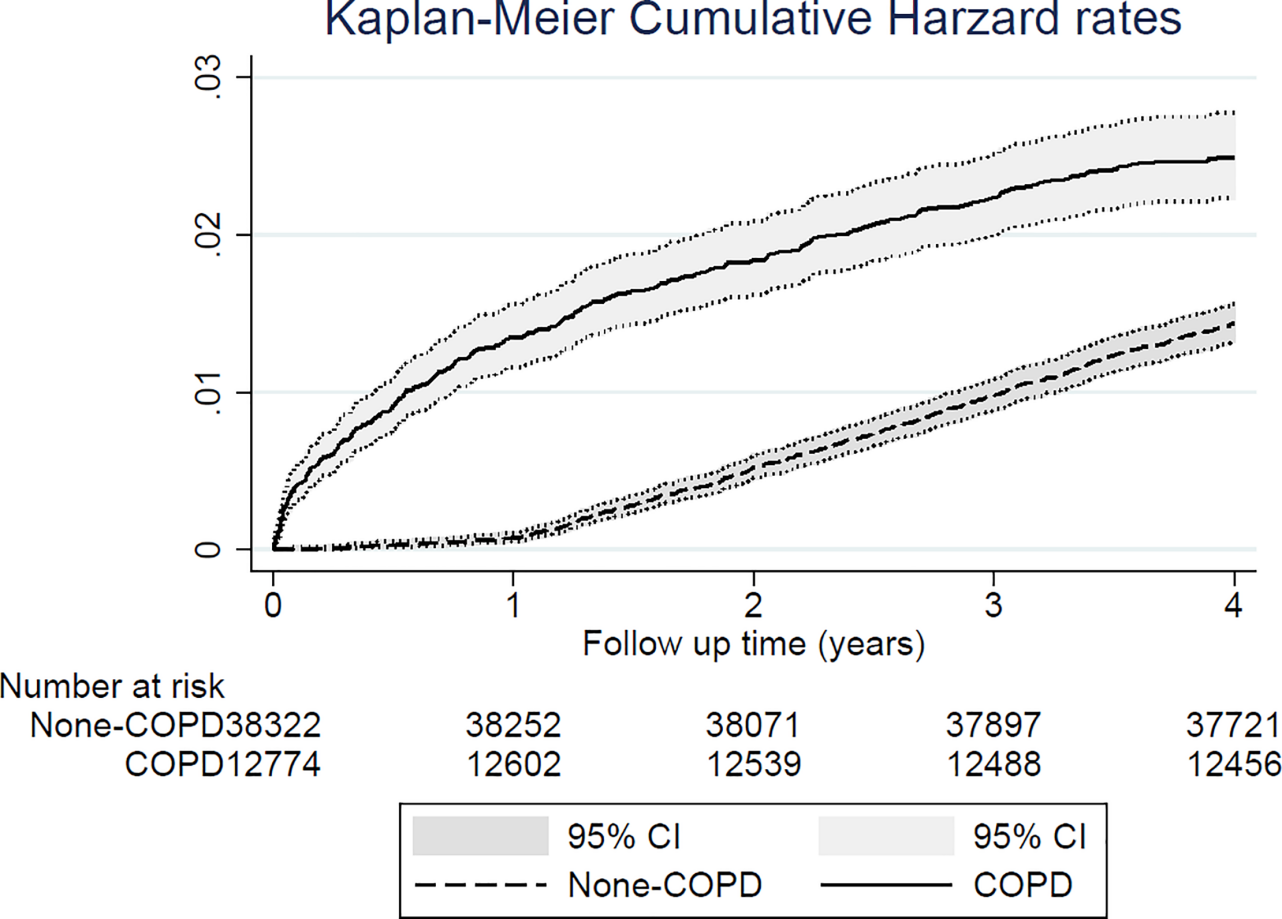
Kaplan–Meier plot of hazard rates for prostate cancer in patients with COPD and gout and controls during the follow-up period of up to 4 years.

**Fig 4:**
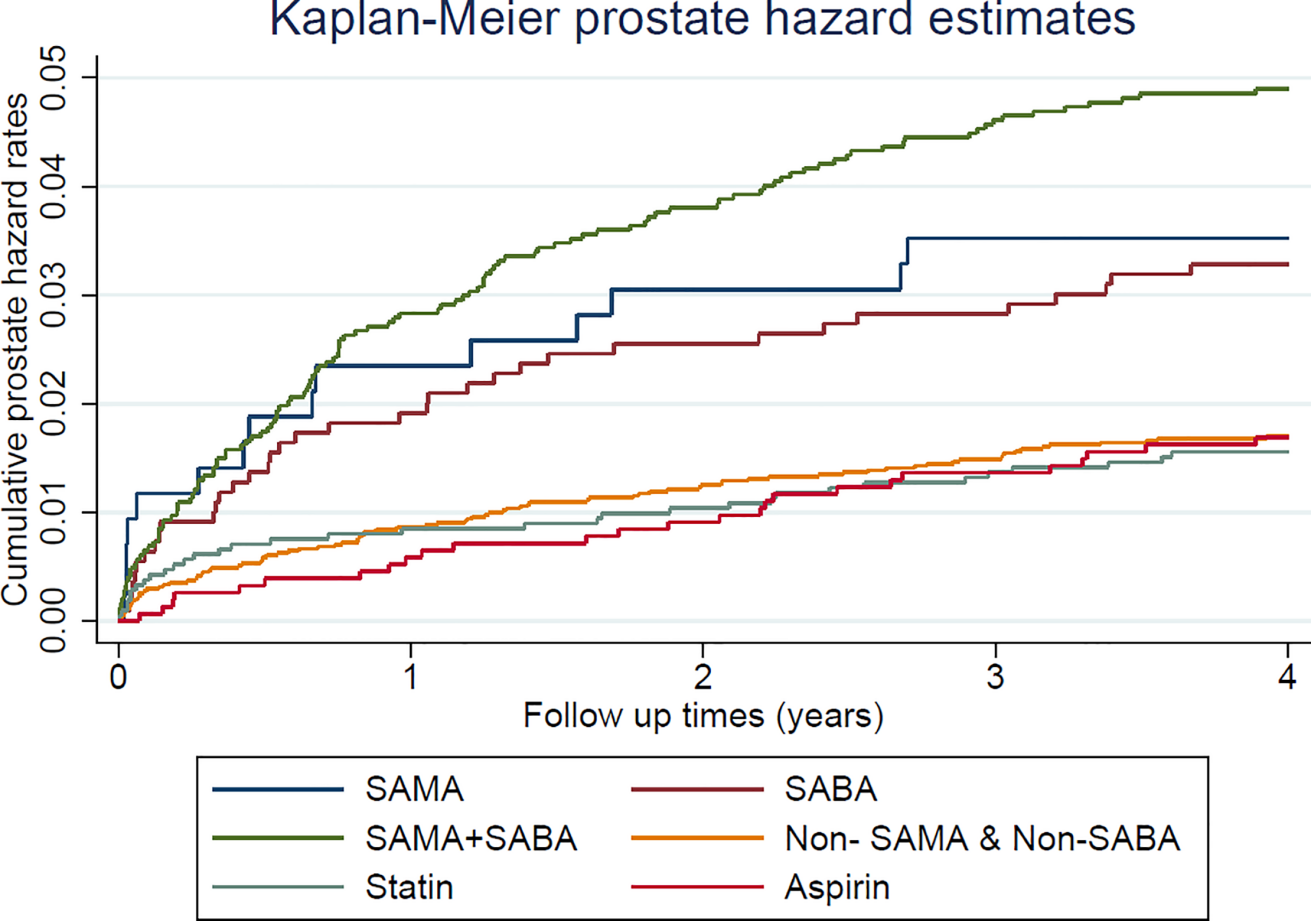
Kaplan–Meier plot of hazard rates for prostate cancer of COPD patients with SABA, SAMA, aspirin and statin medication with up to 4 years of following period.

As indicated by the LHID, the prevalence of comorbidities such as myocardial infarction (2.3%), congestive heart failure (16.3%), peripheral vascular disease (7.0%), and cerebrovascular disease (25.3%) was higher in the COPD cohort than in control cohort. Moreover, the validation database (NHIS) was used to acquire the missing confounders of alcohol drinking, smoking, and BMI in the main database of LHID (Table 1). The incidence of prostate cancer was 633 per 100,000 person-years in the COPD cohort and 361 per 100,000 person-years in the control cohort. Compared with the control cohort, the crude HR was 1.75 (95% confidence interval [CI], 1.52–2.01, p < 0.001), the adjusted HR (adjusted with patients’ age and CCI) was 1.66 (95% CI, 1.44–1.91, p < 0.001), and the propensity score calibration-adjusted HR (adjusted with patients’ age, CCI, and the missing confounders of smoking, alcohol drinking, and BMI) was 1.62 (95% CI, 1.40–1.87, p < 0.001) for the risk of prostate cancer in the COPD cohort (Table 2). Fig 3 displays the Kaplan–Meier hazard curves for the risk of prostate cancer in the COPD and control cohorts during the 4-year follow-up period. The sensitivity analysis of Table 3 revealed adjusted HR was 7.36 (Bootstrap 95% CI, 5.55–9.92, empirical p = 0.001) for 1 year of follow-up, 2.58 (Bootstrap 95% CI, 2.13–3.13, empirical p = 0.001) for 2 years of follow-up and 1.84 (Bootstrap 95% CI, 1.55–2.13, empirical p = 0.001) for 3 years of follow-up. Table 4 presents the demographic data and comorbidity prevalence of patients with or without prostate cancer in the COPD cohort. Notably, patients with COPD and prostate cancer were younger than patients with COPD but without prostate cancer, and as expected, patients with COPD and prostate cancer had a higher prevalence of metastatic cancer. Furthermore, the multivariate Cox proportional hazard regression revealed that the HR for prostate cancer was 1.61 (95% CI, 1.19–2.16, p = 0.002), 1.89 (95% CI, 1.40–2.54, p < 0.001), 0.63 (95% CI, 0.45–0.89, p = 0.010), and 0.55 (95% CI, 0.35–0.85, p = 0.008) in patients with COPD using SAMAs, SABAs, statin, and aspirin, respectively (Table 5). The Fig 4 represented the risk of prostate cancer when using different medication among COPD patients by Kaplan-Meier hazard rates.

**Table 1:** Demographic characteristics and Charlson comorbidity index for male patients with COPD and male patients without COPD in the LHID main database and NHIS validation database.

	Main study (LHID)					Validation study (NHIS)				
Variable	COPD patients N = 12,774		Non-COPD patients N = 38,322		P value	COPD patients N = 312		Non-COPD patients N = 804		P value
	No.	%	No.	%		No.	%	No.	%	
Age (years)					1					1
51–60	2361	18.5	7083	18.5		81	26.0	243	30.2	
61–70	3069	24.0	9207	24.0		71	22.8	213	26.5	
>70	7344	57**0**5	22,032	57.5		160	51.3	348	43.3	
Charlson Comorbidity Index (CCI)										
Myocardial Infarction					0.003					0.043
Yes	293	2.3	716	1.9		10	3.2	11	1.4	
No	12,481	97.7	37,606	98.1		302	96.8	798	98.6	
Congestive Heart Failure					<0.001					<0.001
Yes	2082	16.3	2974	7.8		47	15.1	53	6.6	
No	10,692	83.7	35,348	92.2		265	84.9	751	93.4	
Peripheral Vascular Disease					<0.001					<0.001
Yes	895	7.0	1909	5.0		32	10.3	37	4.6	
No	11,879	93.0	36,413	95.0		280	89.7	767	95.4	
Cerebrovascular Disease					<0.001					<0.001
Yes	3237	25.3	7020	18.3		83	26.6	137	17.0	
No	9537	74.7	31,302	81.7		229	73.4	667	83.0	
Dementia					<0.001					0.330
Yes	993	7.8	1698	4.4		17	5.4	33	4.1	
No	11,781	92.2	36,624	95.6		295	94.6	771	95.9	
Connective Tissue Disease-Rheumatic Disease					<0.001					0.222
Yes	506	4.0	914	2.4		12	3.8	20	2.5	
No	12,268	96.0	37,408	97.6		300	96.2	784	97.5	
Peptic Ulcer Disease					<0.001					<0.001
Yes	5122	40.1	10,036	26.2		132	42.3	232	28.9	
No	7652	59.9	28,286	73.8		180	57.7	572	71.1	
Mild Liver Disease					<0.001					0.039
Yes	2956	23.1	6441	16.8		78	25.0	156	19.4	
No	9818	76.9	31,881	83.2		234	75.0	648	80.6	
Diabetes without Complications					<0.001					0.114
Yes	2961	23.2	8292	21.6		78	25.0	166	20.6	
No	9813	76.8	30,030	78.4		234	75.0	638	79.4	
Diabetes with Complications					0.675					0.329
Yes	862	6.7	2545	6.6		15	4.8	51	6.3	
No	11,912	93.3	35,777	93.4		297	95.2	753	93.7	
Paraplegia or Hemiplegia					<0.001					0.479
Yes	235	1.8	514	1.3		7	2.2	13	1.6	
No	12,539	98.2	37,808	98.7		305	97.8	791	98.4	
Renal Disease					<0.001					0.421
Yes	1052	8.2	2585	6.7		21	6.7	44	5.5	
No	11,722	91.8	35,737	93.3		291	93.3	760	94.5	
Moderate or Severe Liver Disease					0.340					0.691
Yes	50	0.4	128	0.3		1	0.3	4	0.5	
No	12,724	99.6	38,194	99.7		311	99.7	800	99.5	
Metastatic Carcinoma					0.663					0.163
Yes	78	0.6	221	0.6		4	1.3	4	0.5	
No	12,696	99.4	38,101	99.4		308	98.7	800	99.5	
AIDS/HIV					0.004					Na
Yes	9	0.1	7	0.0		0	0	0	0	
No	12,765	99.9	38,315	100.0		312	100	804	100	
CCI Score (SD)	1.7	(1.5)	1**·**2	(1.3)	<0.001	1.7	(1.4)	1.2	(1.3)	
Smoking										0.012
Yes						186	59.6	412	51.2	
No						126	40.4	392	48.8	
Alcohol Drinking										0.151
Yes						107	34.3	313	38.9	
No						205	65.7	491	61.1	
BMI (SD)						23.4	(3.7)	24.2	(3.9)	0.002

COPD, chronic obstructive pulmonary disease

**Table 2:** Crude and adjusted hazard ratios for prostate cancer in patients with COPD and patients without COPD during the 4-year follow-up period (n = 51,096).

Presence of prostate cancer	Non-COPD	COPD	P value
Yes/Total	550/38,322	318/12,774	< 0.001
Incidence (100,000 person-years)	361	633	< 0.001
Crude HR (95% CI)	1.00	1.75(1.52–2.01)	< 0.001
Adjusted^a^ HR (95% CI)	1.00	1.66(1.44–1.91)	< 0.001
Two-stage method adjusted HR (95% CI)^b^	1.00	1.62(1.40–1.87)	< 0.001

^a^Adjustment for propensity score, including patients’ age and Charlson Comorbidity Index (CCI).

^b^Adjustment for propensity score, including patients’ age, Charlson Comorbidity Index (CCI), and the missing confounders of smoking, alcohol drinking, and body mass index.

**Table 3:** Sensitivity analysis—Hazard ratio point estimates and bootstrap 95% CI for prostate between COPD and none-COPD patients.

Presence of prostate cancer	PID		
	Hazard Ratio ^A^	Bootstrap 95% CI	Empirical p-value
1 year of follow-up	7.36	5.55–9.92	0.001
2 year of follow-up	2.58	2.13–3.13	0.001
3 year of follow-up	1.84	1.55–2.13	0.001

^A^ Adjustments are made for patient’s, age, Charlson Comorbidity Index (CCI).

**Table 4:** Characteristics of male patients with COPD with or without prostate cancer.

Variable	COPD cohort (N = 12,774)				p value
	Prostate cancer N = 318		Non-prostate cancer N = 12,456		
	Total no.	%	Total no.	%	
Age (mean ± SD)	70.3 ± 10.26		73.5 ± 8.00		<0.001
Charlson Comorbidity Index (CCI)					
Myocardial Infarction					0.911
Yes	7	2.2	286	2.3	
No	311	97.8	12,170	97.7	
Congestive Heart Failure					0.458
Yes	47	14.8	2035	16.3	
No	271	85.2	10,421	83.7	
Peripheral Vascular Disease					0.776
Yes	21	6.6	874	7.0	
No	297	93.4	11,582	93.0	
Cerebrovascular Disease					0.655
Yes	84	26.4	3153	25.3	
No	234	73.6	9303	74.7	
Dementia					0.430
Yes	21	6.6	972	7.8	
No	297	93.4	11,484	92.2	
Rheumatic Disease					0.450
Yes	10	3.1	496	4.0	
No	308	96.9	11,960	96.0	
Peptic Ulcer					0.148
Yes	140	44.0	4982	40.0	
No	178	56.0	7474	60.0	
Mild Liver Disease					0.197
Yes	64	20.1	2892	23.2	
No	254	79.9	9564	76.8	
Diabetes without Complications					0.715
Yes	71	22.3	2890	23.2	
No	247	77.7	9566	76.8	
Diabetes with Complications					0.434
Yes	18	5.7	844	6.8	
No	300	94.3	11,612	93.2	
Paraplegia or Hemiplegia					0.950
Yes	6	1.9	229	1.8	
No	312	98.1	1227	98.2	
Renal Disease					0.431
Yes	30	9.4	1022	8.2	
No	288	90.6	11,434	91.8	
Moderate or Severe Liver Disease					0.258
Yes	0	0	50	0.4	
No	318	100	12,406	99.6	
Metastatic Carcinoma					<0.001
Yes	26	8.2	52	0.4	
No	292	91.8	12,404	99.6	
AIDS/HIV					0.632
Yes	0	0	9	0.1	
No	318	100	12,447	99.9	

**Table 5:** Multivariate Cox proportional hazard regression for the presence of prostate cancer in male patients with COPD.

Presence of prostate cancer	HR	95% CI	p value
SAMA	1.61	1.19–2.16	0.002
SABA	1.89	1.40–2.54	<0.001
LAMA	0.94	0.63–1.39	0.763
LABA	0.95	0.35–2.58	0.924
LABA plus ICS	0.87	0.64–1.19	0.399
Statin	0.63	0.45–0.89	0.010
Aspirin	0.55	0.35–0.85	0.008
Steroid	0.80	0.60–1.06	0.127
NSAID	1.01	0.65–1.57	0.950
CCI score	0.97	0.89–1.06	0.458
Age (continuous)	1.02	1.00–1.03	0.008

SAMA, short-acting muscarinic antagonists. SABA, short-acting beta-agonists. LAMA, long-acting muscarinic antagonists. LABA, long-acting beta-agonists. ICS, inhaled corticosteroids

## Discussion

Our population-based, retrospective, and longitudinal cohort study demonstrated that male patients with COPD had a 1.62-times higher risk of prostate cancer than those without COPD. Further analysis revealed that patients with COPD who regularly used SABAs or SAMAs had a higher risk of prostate cancer than those who did not use medications. In contrast to short-acting bronchodilators, statin and aspirin could lower the risk of prostate cancer in patients with COPD.

Previous studies have investigated the association of COPD with a wide spectrum of cancer risk.[14, 27] For example, Chiang et al. analyzed 50,875 patients with COPD and found increased risks of incident cancers, including lung and mediastinal cancer, head and neck cancer, breast cancer, prostate cancer, central nervous system (CNS) cancer, lymphoma, and multiple myeloma. They also found that patients with COPD had a 1.2-times higher risk of prostate cancer.[14] Similarly, Ho et al. investigated the risk of cancer in 13,289 patients, and demonstrated higher risks of lung, liver, colorectal, breast, prostate, and stomach cancers in those patients with COPD than in those patients without COPD.[27]

Nevertheless, the definitive mechanism of carcinogenesis in patients with COPD is still subject to debate. Previous research has indicated that chronic inflammatory status and hypoxia-induced oxidative stress can lead carcinogenesis.[28–30] Moreover, chronic inflammation and hypoxic status have been observed in patients with COPD.[11, 31, 32] Therefore, we hypothesized that these factors could be one of the possible mechanisms of carcinogenesis in patients with COPD. Prior scholarship has also indicated that both prostate-localized inflammation and systemic inflammation can induce the carcinogenesis of prostate cancer.[19–21] In contrast to inflammation, which can put patients with COPD at risk for prostate cancer, the endocrinology aspect may be a protective factor in male patients with COPD. Previous studies have noted that androgens are required for prostate gland growth and development, and are considered to be key risk factors for prostate cancer.[33, 34] Another recent cross-sectional study compared 69 male patients with COPD with 82 healthy volunteers, and found a significant reduction in total and free testosterone in the patients with COPD.[35] However, this finding is inconsistent with our study results. Similarly, one previous study investigated the association between endogenous sex hormones and prostate cancer by analyzing 18 prospective studies, and indicated no association between testosterone and the risk of prostate cancer.[36] Therefore, although the testosterone level is lower in patients with COPD, they still have a higher risk of prostate cancer, and the influence of testosterone on prostate cancer remains controversial.

Our study analyzed the inhaled medication used by patients with COPD and demonstrated that short-acting bronchodilators, such as SABAs and SAMAs, can increase the risk of prostate cancer in patients with COPD. Short-acting inhaled bronchodilators are often taken as rescue medicine for the acute exacerbation of COPD. Our study also revealed a higher risk of prostate cancer in patients with COPD who use two types of short-acting bronchodilators (Fig 4). However, long-acting bronchodilators, which exhibit the same action mechanism as that of β-agonists (LABAs) and muscarinic antagonists (LAMAs), did not increase the risk of prostate cancer in patients with COPD. According to the assessment of exacerbation history and the status of airflow limitation, inhaled long-acting bronchodilators along or combined with ICSs are prescribed to patients with moderate to severe COPD.[2] Moreover, two large-scale, randomized, double-blind clinical trials demonstrated that long-acting bronchodilators with or without inhaled steroids can improve pulmonary function and quality of life, and decrease the acute exacerbations of COPD during the 4-year follow-up period.[37, 38]

Long-acting inhaled bronchodilators modulate the inflammation process in patients with COPD; such modulation may be a reason for the lower risk of prostate cancer in patients with COPD who use long-acting bronchodilators, compared with those who use short-acting bronchodilators. Short-acting bronchodilators are often prescribed alone for less severe COPD cases and are taken to relieve COPD symptoms. However, they cannot relieve the inflammatory status in patients with COPD, and the chronic systemic inflammatory characteristics may increase the risk of prostate cancer. Previous studies have found that the use of long-acting bronchodilators with inhaled steroids can decrease systemic inflammation, as demonstrated by decreased C-reactive protein (CRP) and interleukin-6 levels in patients with COPD.[39, 40] We hypothesized that inflammation control in patients with COPD may be crucial for protecting such individuals against subsequent prostate cancer development.

In recent years, increasing evidence has demonstrated that statin use reduces the risk of prostate cancer.[41] Cell and animal models have been used to investigate the molecular mechanisms through which statins inhibit the development of prostate cancer. One prior study investigated the immunomodulatory effects of statin, and found that statins suppressed the inflammatory process of CNS, which may be a protective mechanism through which statins lower the risk of prostate cancer.[42] Statins can also inhibit the angiogenesis and adhesion of prostate cancer cells and induce the apoptosis and cell growth arrest of these cells.[42–44] These findings support the present study results, which revealed that patients with COPD using statin had a lower risk of prostate cancer. Our study also found that COPD patients using aspirin had a lower risk of prostate cancer. A systemic review and meta-analysis found that aspirin can decrease prostate cancer incidence and mortality,[45] and another previous study demonstrated that the anti-inflammatory and antiplatelet effects of aspirin facilitate cancer prevention.[46] One recent report indicated that overexpression of prostaglandin and the cyclo-oxygenase enzyme could induce prostate cancer.[47] We hypothesized that aspirin can inhibit the cyclo-oxygenase enzyme, and therefore decrease the risk of prostate cancer in patients with COPD.

The strength of this study is in its large sample size, and the fact that it acquired the missing confounders (alcohol drinking, smoking, and BMI) from the NHIS. These confounders are important for patients with COPD and have not been presented by other NHIRD research in Taiwan. Nevertheless, this study has some limitations. First, similar to most NHIRD research, the diagnoses of COPD and prostate cancer were determined by clinicians; thus, the diagnostic accuracy should be verified. To correctly identify patients with COPD for their inclusion in the study cohort, we only enrolled patients with at least 3 consecutive claims records of related pharmacotherapy. Moreover, the National Health Insurance Administration in Taiwan randomly and regularly assigns specialists to review medical charts to verify diagnostic accuracy and correlated medical payment. No laboratory data on prostate-specific antigen are available, and imaging studies and histology findings cannot be obtained from the database. However, the coding for prostate cancer is strictly evaluated because prostate cancer coding indicates that patients have a catastrophic illness; thus, any prostate-cancer-related medical expenditure is waived. Second, the severity of COPD and patients’ prostate cancer stages could not be represented in this study. The pulmonary function test data of patients with COPD were limited; thus, it was difficult to determine the association of COPD severity with the risk of prostate cancer. Further investigation of the stratified severity of COPD and the risk of prostate cancer is warranted. Third, we didn’t enrolled COPD patients with multi-inhalation medication with consideration of drug and drug interaction when analyzing the risk of prostate cancer. These patients could be more severe than participants in study cohort and at higher risk of other co-morbidities such as cancer. Finally, medication used by COPD patients was determined using prescription claims from the database, and information on medication compliance is not available in this database. To accurately analyze the effect of COPD medication on the risk of prostate cancer, only patients with at least three records of prescription claims without changes in inhaled medication within 1 year were included in the study cohort. We considered these patients to have adhered to their medication prescriptions, indicating a reliable medication compliance in this study.

## Conclusions

Our large-scale, retrospective, case–control cohort study demonstrated that patients with COPD are at a higher risk of prostate cancer, particularly those using SAMAs or SABAs. We hypothesized that chronic inflammation may be a predisposing factor for the pathogenesis of prostate cancer. Our study also found that aspirin and statin could decrease the risk of prostate cancer in patients with COPD. This finding implies that inflammation control may decrease the risk of prostate cancer. Controlling chronic inflammation in patients with COPD may be a key prevention factor for prostate cancer. Further comprehensive prospective research of the correlation between COPD severity and the risk of prostate cancer, and prognosis with medication, is recommended.
